# Self, peer, and teacher reports of victim‐aggressor networks in kindergartens

**DOI:** 10.1002/ab.21817

**Published:** 2019-01-24

**Authors:** Gijs Huitsing, Marijtje A. J. van Duijn, Tom A. B. Snijders, Françoise D. Alsaker, Sonja Perren, René Veenstra

**Affiliations:** ^1^ University of Groningen Groningen The Netherlands; ^2^ University of Oxford Oxford UK; ^3^ University of Bern Bern Switzerland; ^4^ University of Konstanz Konstanz Germany; ^5^ Thurgau University of Teacher Education Kreuzlingen Switzerland

**Keywords:** aggression and victimization relations, early childhood, exponential random graph models, informants, social networks

## Abstract

This study investigated if and how children and teachers differ in their assessment of victim‐aggressor relationships in kindergartens. Self‐, peer, and teacher reports of victimization‐aggression networks (*who is victimized by whom*) were investigated in 25 Swiss kindergartens with 402 5‐ to 7‐years‐old. It was examined whether child characteristics (sex and parent‐reported internalizing and externalizing behavior) influence informant reports of victimization and/or aggression. Findings from statistical network models indicated higher concordance between self and peer reports than between one of these and teacher reports. Results further showed more agreement among informants on aggressors than on victims. Aggressors reported by self and peer reports were low on internalizing behavior, and aggressors reported by self and teacher reports were high on externalizing behavior; teacher‐reported victims were also high on externalizing behavior. Internalizing behavior was unrelated to victimization. According to self and peer reports, boys as well as girls were victimized by boys and girls equally; teachers reported less cross‐sex victimization than same‐sex victimization. The different views of teachers and children on victim‐aggressor relationships have implications for the identification of aggression in early childhood. Mutual sharing of information between children, their parents, peers, and teachers may contribute to signaling victims and aggressors in the early school years.

## 1 INTRODUCTION

The early school years are important for children's socialization and psychosocial well‐being (Gower, Lingras, Mathieson, Kawabata, & Crick, 2014). When children enter kindergarten, they participate in a peer group and learn to cope with a new environment. In kindergartens, difficulties in social interactions in a natural setting can be detected early on by professionals. Identifying and addressing behavioral problems at a young age may prevent escalation in later years (Barker et al., 2008). Differences between children, teachers, and parents in observing victimization may hinder the early identification of at‐risk children. It is, therefore, important to gain more knowledge about different informant perspectives on victimization in the early school years.

The aim of the present research was to investigate whether and how children and teachers differ in the recognition of victim‐aggressor relationships in kindergartens. A multi‐informant approach was used to investigate the perspectives of children themselves (self‐reports), their classmates (peer reports), and their teachers (teacher reports) on who was victimized by whom. We investigated the pairwise concordance of self, peer, and teacher reports, in order to determine to what extent informants agreed in their judgment of victims and aggressors, including “who is victimized by whom.” Moreover, we investigated whether children's sex and internalizing and externalizing behavior (reported by parents) was related to the informants’ reports, thereby determining whether these child characteristics influenced the extent to which informants reported victimization and/or aggression.

### 1.1 Informants of victimization and aggression

A challenge for research on negative peer relations is how such sensitive data can be obtained reliably (Clemans, Musci, Leoutsakos, & Ialongo, 2014; Ladd & Kochenderfer‐Ladd, 2002): who should be used as informants about children's victimization relations? Different informants provide different views on children's social, emotional, and behavioral problems (De Los Reyes & Kazdin, 2005).

The reliability of information about victimization and aggression is related to the context in which it occurs and the competence of the informant to report information (Ladd & Kochenderfer‐Ladd, 2002). An advantage of self‐reports is that they capture specific experiences of victims not observable by others. A disadvantage is potential bias, because children may provide socially desirable answers, may not be willing to report painful experiences, or may overreport victimization and underreport aggression (Monks, Smith, & Swettenham, 2003). An advantage of peer reports is that many raters in a classroom assess the behavior of a classmate. Peers may be less subject to underreporting bias, given that they are part of the peer group and often present during incidents of aggression, even in unsupervised contexts (O'Connell, Pepler, & Craig, 1999). Peers may be willing to report the painful experiences of others. However, peers may also be sensitive to prejudices and reputational effects. Young children may be more subject to over‐ or underreporting biases, because of difficulties in differentiating aggression from other negative peer interactions. Teachers have regular opportunities to observe children in the classroom, although they have a different position to observe victimization than peers. Teachers may not always be aware of victimization incidents, because they may not be present during their occurrence (when, in contrast, peers are often present), and are not necessarily informed by victims or other witnesses (Neal, Cappella, Wagner, & Atkins, 2011). Teachers draw finer, qualitative distinctions between children's behaviors than children themselves, especially compared with young children.

In one study, student‐teacher agreement on victim‐aggressor relations in 38 American classrooms (6‐ to 11‐years‐old) was rather low, with on average only 8% of victim‐aggressor relations reported by both the teacher and student (Ahn, Rodkin, & Gest, 2013). Student‐teacher agreement was almost twice as high for same‐sex victimization and boys victimizing girls than for girls victimizing boys. Investigations into concordance between informants in early childhood showed that agreement between children, peers, and teachers was higher for bullies or aggressors than for victims (Camodeca, Caravita, & Coppola, 2015; Lee, Smith, & Monks, 2016; Monks et al., 2003; Perren & Alsaker, 2006). An explanation might be that aggression is usually more stable in early childhood than victimization (Murray‐Close & Ostrov, 2009; Snyder et al., 2003).

### 1.2 Involvement in victimization and aggression by sex and internalizing and externalizing behavior

Sex is an important characteristic that is associated with involvement in victimization and aggression; boys are often more aggressive than girls (Hong & Espelage, 2012). In early childhood, there is a general tendency for children to have sex‐segregated peer groups because of the different play styles of boys and girls (Cherney & London, 2006). This opportunity structure makes it reasonable to expect that victimization would occur often within same‐sex relations (Crick et al., 2006). However, studies have shown that cross‐sex victimization occurs in early childhood, with boys having both male and female targets, rather than girls targeting boys (Hanish, Sallquist, DiDonato, Fabes, & Martin, 2012; Veenstra, Verlinden, Huitsing, Verhulst, & Tiemeier, 2013); this is in line with victim‐bully relations in late childhood and preadolescence (e.g., Veenstra et al., 2007).

Internalizing and externalizing behavior is associated with involvement in victimization and aggression (Neal, Durbin, Gornik, & Lo, 2017). Externalizing behavior, such as aggression toward specific peers or disobedient behavior, is disruptive outward behavior to the external environment and has been found to be related to peer‐ and teacher‐reported peer aggression (Perren & Alsaker, 2006). Moreover, externalizing behavior is strongly related to victimization in early childhood (Arseneault et al., 2006; Hanish & Guerra, 2002; Perren, Von Wyl, Stadelmann, Burgin, & Von Klitzing, 2006; Snyder et al., 2003). An explanation is that young children may retaliate to aggression (Hanish et al., 2012). Internalizing behavior, such as withdrawn, introvert, anxious, or depressed behavior, is related to victimization in early childhood (Hanish & Guerra, 2002; Leadbeater & Hoglund, 2009; Perren et al., 2006; Van Lier et al., 2012).

### 1.3 The present study

Investigations into victimization and aggression often classified children into the “role” of aggressor, victim, or aggressive victim. The current study takes a step further by investigating victim‐aggressor relationships using a social network perspective: *who is victimized by whom*? Victimization and aggression are relational phenomena where children can be involved in multiple relations (Huitsing et al., 2012). A special class of statistical models for social network analysis (Exponential Random Graph Models or ERGMs, see Lusher, Koskinen, & Robins, 2013) enables the investigation of the structure and interdependencies of the multiple relationships to, from, and between children, the effects of individual and dyadic characteristics, and agreement between two informants.

Concordance on victim‐aggressor relations was expected to be higher between self and peer reports than between child and teacher reports, because teachers observe victimization and aggression from a different position than children (*H1*). Moreover, it was expected that concordance between the three informants would be higher for aggressors than for victims (*H2*), given that aggression is more visible and stable than victimization in early childhood. We further expected more reporting of same‐sex victimization than cross‐sex victimization; in case of cross‐sex victimization, boys were expected to be aggressors more often than girls (*H3*). The reports of informants was expected to associate externalizing behavior with both victimization and aggression (*H4a*), whereas internalizing behavior was expected to be associated exclusively with victimization (*H4b*). In addition, we explored differences in internalizing and externalizing behavior in victim‐aggressor relationships; it was predicted that aggressors would have more externalizing behavior than their victims (*H5a*), and that victims would have more internalizing behavior than their aggressors (*H5b*).

## 2 METHOD

### 2.1 Sample and participants

This study used the pre‐test data of the prevention program *Pathways to Victimization* (Alsaker & Valkanover, 2012). For the present study, we used a subsample for which teachers and parents filled out in‐depth questionnaires. Data stemmed from 402 children in 25 kindergartens (collected from December 2004 to January 2005). The participation rate was high; only 2.5% of the parents refused participation for their child. Overall, the mean age in the sample was 5.8 years (SD = 0.58). A more extensive description of the sample, sampling procedure, and participants can be found in Appendix S1.

### 2.2 Procedure

The assessment included teacher and parent questionnaires and child interviews. Teachers and parents completed a questionnaire for each child, including items related to behavior in the peer group as well as various behavioral and personality characteristics. Additionally, each child was interviewed individually by trained students. Time was taken to familiarize the children with the procedures and to explain the reasons for the interviews. For example, about one week before the interview, the interviewers visited the kindergarten groups and told a story about “human researchers” who wanted to do research in a kindergarten. The children could ask questions and practice the interview in a role‐play. The interview itself began with the children identifying their peers in photographs.

### 2.3 Victimization networks

#### 2.3.1 Self‐reports

Victimization was explained in an age‐appropriate way by presenting four pictures describing several forms of aggression (i.e., verbal, material, physical, relational) that together represent general aggression (in line with Perren & Alsaker, 2006). Children were asked if they were victimized. If they confirmed, they were asked “By whom are you victimized?” Children could indicate their aggressors by pointing out the pictures of classmates (there was no maximum). These nominations were used to construct networks with victim‐aggressor relations. In this way we obtained information on the perspective of self‐reported victims (given nominations) and their aggressors (received nominations).

#### 2.3.2 Peer reports

Using the same pictures as used for the self‐reports, children were asked to nominate classmates who victimized other children. Children were asked to indicate aggressors by pointing out pictures of classmates. If children nominated aggressors, they were asked to identify the children who were victimized by them. In addition to specifying victims, children were also allowed to indicate whether the aggressor victimized “everybody,” “all the girls,” or “all the boys.”

We considered peer‐reports of victimization‐networks to be meaningful for our research purposes only if children nominated specific victims. Peers provided in total 720 nominations for aggression (this number includes overlapping nominations—some children were nominated by several peers for aggression). For 72.1% of these nominations, children were able to indicate specific victims. For the other cases, peers reported that everyone was victimized by that aggressor (17.4%), or that the aggressor victimized all the girls (2.1%) or all the boys (1.1%). Some peers were not able to report who was victimized (7.4%). Children who were nominated for victimizing everyone (*N* = 68) were more often mentioned as aggressor through specific nominations (*M* *= *4.4) than children who were not nominated for victimizing everyone (*M* *= *1.4), *t*(80) = 7.72, *p *< .01. This suggests that unspecified reports (i.e., victimizing everyone) were also captured by specific reports of other peer reporters. Therefore, we decided not to use the unspecified reports when constructing the networks.

In total, 223 children (55.5% of the sample) were nominated at least once as aggressor; altogether they were involved in 769 victim‐aggressor relations (which is 12.4% of the total number of possible relations in the 25 kindergartens, which is 6,212). The majority of these relations were reported by only one peer (83.0%); 13.8% were reported by two peers, and it rarely occurred that a victim‐aggressor relation was reported by three peers (3.3%; three was the maximum). Using these nominations, peer‐reported networks of victimization were constructed when at least one peer reported a victim‐aggressor relation.

#### 2.3.3 Teacher reports

As part of an extensive questionnaire for each child separately, teachers were asked if the children were aggressive, either verbally, materially, physically, or relationally (similar to peer reports; based on Perren & Alsaker, 2006). If teachers reported that children were aggressive, they were asked to indicate who were victimized by these children: that is, to mention specific victims for each aggressor. Similarly, teachers were also asked which children were victimized and by whom (see further Appendix S1). These nominations were combined; if a teacher reported a victim‐aggressor relation in at least one of the two questions, the victim‐aggressor relation was regarded as present in the networks. In network research, it is a common procedure to collect information on specific relationships with one or two questions, also regarding teacher‐reports on victim‐aggressor relationships (Ahn et al., 2013; Monks et al., 2003).

### 2.4 Parent‐reported internalizing and externalizing behavior

Children's internalizing behavior was measured with a 9‐item scale, derived from the Child Behavior Scale (Ladd & Profilet, 1996). Parents responded on a 4‐point Likert‐type (1 = completely false, 4 = completely true) scale to items that tap internalizing behavior, anxiety, and depression, such as “He/she is often sad” or “He/she is easily frightened.” The scores for the nine items formed a reliable scale and were averaged (Cronbach's *α* = .72).

Children's externalizing behavior was measured with an 8‐item scale, derived from the Child Behavior Scale (Ladd & Profilet, 1996), measured. Parents responded on a 4‐point Likert‐type (1 = completely false, 4 = completely true) scale to items that tap open aggression, verbal aggression, and oppositional defiant behavior, such as “He/she is physically aggressive (hits, kicks, bites)” or “He/she insults other children or shouts at them.” The scores for the eight items formed a reliable scale and were averaged (Cronbach's *α* = .82). More information can be found in Appendix S2.

#### 2.4.1 Missing data imputation

Parental information on internalizing and externalizing behavior was available for 60.7% of the children. To handle the missing data, we performed multiple imputation at the scale level using the MICE package implemented in the R‐system (Van Buuren & Groothuis‐Oudshoorn, 2011) with sex, age, and self, peer, and teacher reported victimization and aggression as predictors to obtain five complete datasets. Simple *t*‐tests on self, peer, and teacher reports showed that children with missing parental data neither received nor gave more nominations for victim‐aggressor relationships. However, as only German and French versions of the questionnaire were offered, non‐responders were more often parents with a migrant background (i.e., one of the parents not originally from Switzerland), *t*(317) = 6.57, *p* < .01. As a result of the imputations, we were able to include data on all children and analyze complete networks.

### 2.5 Analytical strategy

We first examined the descriptive statistics of the network data at the dyadic, individual, and classroom levels. We also inspected the concordance between self, peer, and teacher reports of specific victim‐aggressor relations using the Jaccard similarity index as an indication of the amount of agreement (to provide a first test of *H1* and *H2*). The Jaccard index gives the proportion of agreement in the reports of the present relationships for two informants (Neal et al., 2011, see also footnote C of Table 2). We computed the Jaccard index at the dyadic level, where two informants had to mention the same victim‐aggressor relation, and at the individual level, where both informants needed to mention a child as victim (or aggressor), but not necessarily with the same aggressor (or victim). As an example, a Jaccard index of .5 indicates that of all instances reported by either informant, 50% are reported by two informants.

#### 2.5.1 Statistical network modeling and meta‐analysis

To investigate the agreement between two informants, pairs of networks were analyzed using bivariate Exponential Random Graph Models (Lusher et al., 2013), estimated with *XPNet* (freely available available at www.melnet.org.au/). We had to exclude some classrooms from the estimations, because too few nominations were given in these classrooms for either type of informant. As a consequence, no ERGMs could be estimated. To facilitate comparisons between models, we only present results of the classrooms where all models could be estimated (*N *= 18 with 292 students). Thus, the results can only be generalized to classrooms with a reasonable number of victim‐aggressors relationships reported (in our data: 10 relationships).

The results of the models for the five imputed data sets for each of the three pairs of informants in each classroom were combined according to Rubin (1987). The resulting adjusted parameter estimates and standard errors were summarized in a meta‐analysis. The obtained parameter estimates represents the overall (weighted) mean estimates between classrooms (along with a standard error), accompanied by an estimate of the standard deviation representing variation of the estimations between classrooms. More information on the ERGMs and meta‐analysis can be found in Appendix S3.

#### 2.5.2 Model specification

The model specification was formulated in line with usual practices for ERGMs (Lusher et al., 2013; Robins, Pattison, & Wang, 2009). Several univariate structural parameters were included to control for the structure of each network (Huitsing et al., 2012). These structural parameters were needed for well‐fitting models, but were not the main focus of this study; thus, we explain and discuss them in Appendix S4.

The bivariate structural parameters in Table 1 modeled the presence or absence of concordance between informants (to test *H1*). All networks were constructed in such a way that arrows pointed from victims to aggressors: an outgoing arrow from a child represents that this child was reported by children themselves, peers, or teachers as being victimized, and an incoming arrow represents a nomination as an aggressor (see also the graphical representation in Table 1). The *multiplex relation* modeled at the relational level whether two informants mentioned the same victim‐aggressor relation (i.e., complete agreement) and tests *H1*. *H1* was further tested using the following four bivariate parameters for the three pairs of informants. The *multiplex in‐nominations* modeled agreement of informants on aggressors (irrespective of who the victims were), and the *multiplex out‐nominations* modeled agreement of informants on victims (irrespective of who the aggressors were). These multiplex in‐nominations and out‐nominations also tested *H2*. The parameters for the *mixed nominations* investigated contrasting reports, by modeling whether one informant mentions a child as an aggressor, whereas the other informant mentions the child as a victim, and vice versa.

**Table 1:** Modeling agreement: structural parameters in the multivariate exponential random graph models for victimization networks

Parameter (statistic)	Description	Graphical representation
Multiplex relation (Arc‐AB)	Complete agreement on a nomination for the same victim‐aggressor relation in both network A and B	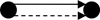
Multiplex in‐nominations (In‐star‐AB)	Agreement of informants on the receiver of a nomination (aggressors) irrespective of the sender of that nomination (victims)	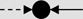
Multiplex out‐nominations (Out‐star‐AB)	Agreement of informants on the sender of a nomination (victims) irrespective of the receiver of that nomination (aggressors)	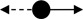
Multiplex mixed nominations AB (Mixed‐star‐AB)	Contrasting reports on children's status: Informant A mentions the child as an aggressor (dotted line) whereas informant B mentions the child as a victim (straight line)	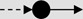
Multiplex mixed nominations BA (Mixed‐star‐BA)	Contrasting reports on children's status: Informant A mentions the child as a victim (dotted line) whereas informant B mentions the child as an aggressor (straight line)	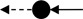

Characters in brackets indicate the names of the parameters as they are named in *XPNet*, where A (dotted lines) refers to one network (i.e., self, peer, or teacher reported victimization networks) and B (straight lines) refers to another network.

Sex was used as a dyadic covariate in the network models to test *H3*. Boy–girl and girl–boy relations were combined into cross‐sex relations to have enough cases in each category to estimate the network models. Boy‐boy relations and cross‐sex relations were compared with girl‐girl relations (the reference category). For internalizing and externalizing behavior, *victim* and *aggressor* effects were included to examine whether internalizing and externalizing behavior was associated with victimization or aggression (*H4a* and *H4b*). The *absolute difference effect* included whether the absolute difference between two children with respect to internalizing or externalizing behavior had an additional effect on the presence of victim‐aggressor relations (*H5a* and *H5b*).

## 3 RESULTS

### 3.1 Descriptive results

Descriptive statistics for the networks are given in Table 2 for the relational (network), individual (child), and classroom levels. The prevalence of victim‐aggressor relationships was highest for peer reports (12.4%) and lowest for victims’ self‐reports (8.4%). The sex composition of these victim‐aggressor relationships was quite similar for self and peer reports. Boys were reported to victimize others more than girls, with boy‐boy victimization relations occurring at a similar rate to girl‐boy relations (girl‐boy indicates that a girl is victimized by a boy), although victimization by boys was reported more often through peer‐reports than through self‐reports. Girls victimized girls and boys to a similar extent (similar proportions of girl–girl and boy–girl relations). The pattern for teacher‐reported victim‐aggressor relations was different. Teachers reported quite similar levels of victimization among boys and among girls (with girl–girl victimization more than twice as high as obtained through self and peer reports), whereas the level of cross‐sex victimization (the combination of girl–boy and boy–girl relations) was less than half that of same‐sex victimization. The Jaccard index on victim‐aggressor relationships was higher between self and peer reports (25%) than between peers and teachers (14%) and between self and teacher reports (11%). The higher agreement between self and peer reports supports *H1*.

**Table 2:** Descriptive statistics of self, peer, and teacher reports on victim‐aggressor relations

	Self‐reports	Peer reports	Teacher reports
**Relation/network level**			
Prevalence (density)^a^	519 (8.4%)	769 (12.4%)	692 (11.1%)
Sex composition^b^			
Girl–girl	72 (5.3%)	98 (7.3%)	192 (14.3%)
Girl–boy	176 (11.2%)	264 (16.8%)	107 (6.8%)
Boy–girl	83 (5.3%)	96 (6.1%)	96 (6.1%)
Boy–boy	188 (11.0%)	311 (18.1%)	297 (17.3%)
Jaccard index^c^			
Self‐report	–		
Peer report	.25	–	
Teacher report	.11	.14	–
**Individual level**			
Jaccard index^c^, ^d^			
Self‐report	–	.59	.44
Peer report	.56	–	.45
Teacher report	.42	.55	–
Average incoming/outgoing nominations per child (in/outdegree)	1.3	1.9	1.7
Standard deviation outgoing nominations for victimization (outdegree)	1.8	1.7	2.2
Standard deviation incoming nominations for aggression (indegree)	1.7	2.7	2.2
**Classroom level**			
Average percentage of aggressors (sinks)^e^ (standard deviation)	22% (12%)	8% (7%)	11% (8%)
Average percentage of victims (sources)^e^ (standard deviation)	20% (12%)	29% (14%)	14% (11%)
Average percentage of isolates^e^ (standard deviation)	24% (19%)	16% (19%)	32% (29%)
Average percentage of aggressive victims^e^ (standard deviation)	35% (22%)	47% (22%)	43% (30%)
Reciprocity for aggression (standard deviation)	15.4% (13.1%)	20.4% (13.5%)	38.1% (27.5%)

^a^ The density is the number of victim‐aggressor relations, relative to the total number of possible relations (6,212).

^b^ The first person in the victim‐aggressor relation is the victim, the second person in the relation is the aggressor (i.e., boy–girl means that a boy is victimized by a girl). The percentages are relative to the total number of possible sex‐relations, which are: girl–girl = 1,346; boy–girl = girl–boy = 1,575; boy–boy = 1,716.

^c^ The Jaccard index is defined by: *N_AB_*/(*N_AB_* + *N_A_* +* N_B_*); *N_AB_* is equal to the relations/individuals reported by both informants, *N_A_* is equal to the relations /individuals reported by informant A, and *N_B_* is equal to the relations/individuals reported by informant B.

^d^ Jaccard indices below the diagonal are for victims, Jaccard indices above the diagonal are for aggressors.

^e^ *Sinks* are children who are mentioned at least once for aggression (at least one incoming nomination) but are not victimized (zero outgoing relations); *Sources* are children who are mentioned at least once for victimization but are not mentioned as aggressors; *Isolates* are children who are reported for neither victimization nor aggression; *aggressive victims* are children who are mentioned both as victims and as aggressors.

Aggregation of victim‐aggressor relations to the individual level showed higher levels of agreement. The Jaccard indices in the second part of the table indicate the agreement between informants with respect to children who were mentioned as victim at least once, and, similarly, with respect to children who were mentioned as aggressor at least once. For example, 56% of the self‐reported victims were also mentioned at least once as victim through peer reports (but not necessarily in the same victim‐aggressor relation). Comparably, 59% of the aggressors reported by victims (through self‐reports) were also nominated by at least one peer. Somewhat higher agreement was found for the aggressors (above the diagonal) than for the victims (below the diagonal; except for the concordance of peer and teacher reports), which is in line with *H2*. Furthermore, peers reported that children were on average victimized by two children, whereas the self‐reported average number of aggressors reported by victims was closer to one. The standard deviation between children was larger for the number of aggressor (incoming) nominations than for the number of victim (outgoing) nominations for peer reports.

At the classroom level, peer reports showed that the majority of the children were involved in victimization networks, either as aggressors (8% of the children were mentioned at least once for aggression but not for victimization), victims (29% of the children were mentioned at least once for victimization but not for aggression), or aggressive victims (47% of the children were mentioned for both victimization and aggression). Only 16% of the children were *isolates*, that is, they were reported neither as victim or aggressor. According to teachers, more than one third of the children in the sample were not victimized (32% isolates plus 11% aggressors) and 43% were reported as aggressive victims. When children themselves were asked about their experiences, 46% of the children did not report being victimized (24% isolates plus 22% aggressors). With regard to received nominations for self‐reports, 57% of the children were mentioned as aggressors. The number of reciprocal relations was relatively high, and highest for teacher reports; about 40% of the teacher‐reported victimization relations were reciprocal.

### 3.2 Network analyses of agreement between informants

The overall results for bivariate network analyses of each pair of informants over the 18 schools are presented in Table 3. This table gives only the parameters for the mutual dependence in the two reported networks, the so‐called multiplex parameters. The complete tables, also containing parameters for univariate structural network effects, are given in Appendix S5.

**Table 3:** “Who is Victimized By Whom?”: bivariate exponential random graph models for network structure of victimization using self, peer, and teacher reports

		Mean parameter		Standard deviation	
Parameter	Statistic	Est.	Std. Err.	Est.	*χ* ^2^
**Self and Peer reports**					
Self‐report and peer report (Arc‐AB)	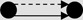	1.93	(0.24)**	0.63	83**
In‐nomination (aggression) self‐report & peer report (In‐star‐AB)	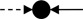	0.22	(0.06)**	0.06	917**
Out‐nomination (victimization) self‐report & peer report (Out‐star‐AB)	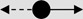	0.11	(0.07)	0.08	467**
In‐nomination self‐report & out‐nomination peer report (Mixed‐star‐AB)	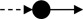	0.23	(0.06)**	0.05	204**
Out‐nomination self‐report & in‐nomination peer report (Mixed‐star‐BA)	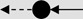	0.05	(0.01)**	0.00	410**
**Self and Teacher reports**					
Self‐report and teacher report (Arc‐AB)	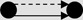	0.96	(0.14)**	0.04	18
In‐nomination (aggression) self‐report & teacher report (In‐star‐AB)	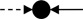	0.18	(0.04)**	0.02	529**
Out‐nomination (victimization) self‐report & teacher report (Out‐star‐AB)	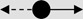	0.04	(0.06)	0.06	355**
In‐nomination self‐report & out‐nomination teacher report (Mixed‐star‐AB)	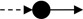	0.13	(0.07)*	0.08	392**
Out‐nomination self‐report & in‐nomination teacher report (Mixed‐star‐BA)	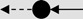	0.10	(0.09)	0.12	543**
**Peer‐ and Teacher reports**					
Peer‐report and teacher report (Arc‐AB)	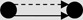	0.93	(0.18)**	0.33	47**
In‐nomination (aggression) peer‐report & teacher report (In‐star‐AB)	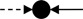	0.10	(0.04)*	0.03	1041**
Out‐nomination (victimization) peer‐report & teacher report (Out‐star‐AB)	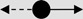	0.07	(0.07)	0.07	414**
In‐nomination peer‐report & out‐nomination teacher report (Mixed‐star‐AB)	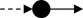	0.06	(0.06)	0.06	1281**
Out‐nomination peer‐report & in‐nomination teacher report (Mixed‐star‐BA)	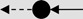	0.03	(0.06)	0.05	561**

The mean parameter is an unstandardized aggregated estimate across classrooms. The standard deviation represents the degree to which estimates vary across classrooms (*N classrooms* = 18). All bivariate analyses also contained uniplex structural parameters, sex, and internalizing and externalizing behavior (see Appendix S5).

*p < .05.

**p < .01.

The multiplex *arc* parameter (*Arc‐AB*) was used to examine whether the same victim‐aggressor relations (i.e., specific victim‐aggressor pairs) were reported by both informants. For all three pairs of informants, this was strongly significant, and the parameter was stronger for self‐peer agreement (*Parameter Estimate* [P.E.] *= *1.93, *p *< .01) than for self‐teacher agreement (P.E. = 0.96, *p* < .01) or peer‐teacher agreement (P.E. *= *0.93, *p* < .01). This result provides support for *H1*. For the first (self‐peer) and the third (peer‐teacher) of these comparisons, the degree of agreement differed between the classrooms (significant “Standard deviation” columns in Table 3).

The multiplex victim and aggressor nomination parameters represent agreement between reporting a child as a victim or aggressor, over and above the agreement on specific victim‐aggressor pairs (i.e., the Arc‐AB effect). For all three informant comparisons, additional agreement was found on aggressors (P.E. for In‐star‐AB: 0.22, *p* < .01; 0.18, *p* < .01; 0.10, *p* < .01). This agreement was not found on victims (Out‐star‐AB); all estimates for multiplex victim nominations were non‐significant. Thus, informants were more concordant with respect to aggressors than to victims, supporting *H2*. Note that, in further support of *H1*, the agreement represented by the multiplex aggressor nominations was higher between the self and peer reports than between the self‐teacher and peer‐teacher reports.

The *mixed nominations* modeled the tendency for one informant to report a child as victim and for the other informant to report the child as an aggressor (and vice versa). For self and peer reports such mixed nominations were found for children who were nominated as aggressors by self‐reported victims and nominated as victims by peer reports (P.E. *= *0.23, *p* < .01); and although weaker, for self‐reported victims to be mentioned by peers as aggressors (P.E. *= *0.05, *p* < .01). Some children who were nominated as aggressors by victims were reported by teachers as victims (P.E. *= *0.13, *p* < .01; middle part of Table 3). No further tendencies toward contrasting reports were found. For each of the multiplex victim, aggressor, and mixed nomination parameters, significant though rather small variation across the classrooms was found.

### 3.3 Sex and internalizing and externalizing behavior related to informants’ reports

The overall results for the effects of sex and internalizing and externalizing behavior in the univariate models of self, peer, and teacher reports are given in Table 4. The full table can be found in Appendix S6.

**Table 4:** “Who is Victimized By Whom?”: univariate exponential random graph models for network structure of victimization with sex and internalizing and externalizing behavior

		Self‐reports				Peer reports				Teacher reports			
		Mean parameter		Standard deviation		Mean parameter		Standard deviation		Mean parameter		Standard deviation	
Parameter	Statistic	Est.	Std. Err.	Est.	*χ* ^2^	Est.	Std. Err.	Est.	*χ* ^2^	Est.	Std. Err.	Est.	*χ* ^2^
**Relational covariates**													
Girl–girl	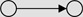	Ref.				Ref.				Ref.			
Cross‐sex	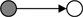	0.00	(0.31)	0.95	55**	0.08	(0.21)	0.44	57***	−1.42	(0.23)***	0.42	58***
	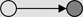												
Boy–boy	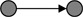	0.01	(0.20)	0.20	24	0.22	(0.18)	0.23	33**	−0.23	(0.21)	0.45	72***
**Individual covariates**													
Internalizing behavior													
Victim	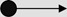	0.20	(0.24)	0.19	15	0.15	(0.19)	0.00	10	0.04	(0.18)	0.15	23
Aggressor	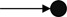	−0.55	(0.20)**	0.00	6	−0.29	(0.11)**	0.05	21	−0.13	(0.13)	0.05	21
Abs. dif.	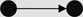	0.22	(0.19)	0.00	7	0.07	(0.13)	0.00	15	0.05	(0.14)	0.01	14
Externalizing behavior													
Victim	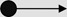	−0.20	(0.18)	0.09	14	−0.06	(0.15)	0.00	11	0.38	(0.16)**	0.08	19
Aggressor	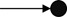	0.56	(0.20)***	0.10	16	0.15	(0.11)	0.09	31**	0.33	(0.19)^†^	0.22	29**
Abs. dif.	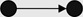	−0.01	(0.11)	0.00	15	0.09	(0.16)	0.16	30**	0.17	(0.17)	0.12	23

The mean parameter is an unstandardized aggregated estimate across classrooms. The standard deviation represents the degree to which estimates vary across classrooms (*N = *18). Abs. dif. = Absolute difference score. The models also contained uniplex structural parameters (see Appendix S6).

^†^ *p* < .10.

**p < .05.

***p < .01.

The first part of Table 4 provides the results for sex, with girl‐girl victimization relations as the reference category. In the self and peer reported networks, neither boy–boy victimization nor cross‐sex victimization occurred significantly more often than girl‐girl victimization which is partly in line with *H3*. Furthermore, in the teacher reported aggression networks, boy–boy victimization was reported as often as girl‐girl victimization. However, in line with the descriptives, cross‐sex victimization was significantly less reported by teachers than same‐sex victimization (P.E. *= *−1.42, *p* < .01). Note that almost all sex effects showed significant variation across the classrooms. This means, for example, for teacher reports, that some teachers reported more boy–boy relations than girl‐girl relations, whereas other teachers reported fewer boy–boy relations than girl–girl relations.

The second part of Table 4 concerns internalizing and externalizing behavior. Externalizing behavior was significantly associated with reports on aggression through self‐reports (P.E. *= *0.56, *p *< .01) and marginally significant through teacher reports (P.E. *= *0.33, *p *< .10). In addition, externalizing behavior was associated with teacher nominations on victimization (P.E. *= *0.38, *p *< .01). These results are partly in line with *H4a*. Internalizing behavior was not found to be associated with reports on victimization (providing no support for *H4b*). Children with internalizing behavior were less likely to be nominated as aggressor through victims’ self‐reports (P.E. *= *−0.55, *p *< .05) and by peers (P.E. *= *−0.29, *p *< .05). For none of the informants significant estimates for *absolute difference scores* were found, suggesting that aggressors did not differ from their specific victims, nor resembled them, with regard to internalizing or externalizing behavior, more than already implied by the *victim* and *aggressor* effects (providing no support for *H5a* and *H5b*).

## 4 DISCUSSION

In this study we investigated reports of three types of informants on victim‐aggressor relationships in kindergartens. Overall, results showed considerable agreement between informants on who is victimized by whom. The network information, however, also demonstrated clear differences in the assessment of victim‐aggressor relationships. Especially teacher reports differed from the perspectives of the self and peer reports, and these differences were partly explained by children's sex and internalizing and externalizing behavior.

### 4.1 Concordance on victim‐aggressor relationships

Prevalence rates varied between informants: self‐reports identified 8% of the possible relationships between children as victim‐aggressor relations, whereas the prevalence was 12% using peer reports and 11% using teacher reports. It is not surprising that peer reports were higher on average; peer reports were aggregated over all peers in the classroom, with a victim‐aggressor relation being reported if it was mentioned by at least one peer.

Agreement on specific victim‐aggressor relationships was relatively low. It was highest between children and their peers, and lower between children and the teacher (in line with *H1*); 25% of the total number of self and peer‐reported victim‐aggressor relations were reported by both. Agreement between peer and teacher reports was lower (14% shared reports), and even lower between self and teacher reports (10% shared reports). At the child level, there was more agreement (compared with the relational level) in being mentioned as an aggressor at least once or being mentioned as a victim at least once. Agreement was in the range of 40–60%. The statistical network models account for the dependencies in the data and shed more light on concordance among informants. Agreement was most strongly expressed in the tendency to report the same victim‐aggressor pairs. In addition, there was a tendency to agree in reporting aggressors without necessarily agreeing on their victims, but no tendency to agree in reporting victims. These findings are in line with *H2* and earlier investigations (Camodeca et al., 2015; Lee et al., 2016; Perren & Alsaker, 2006). The current findings further contributed to knowledge on concordance between informants at the relational level (Ahn et al., 2013). Some results provided evidence for disagreement: a tendency for self‐reported victims to nominate aggressors who were nominated as victims by peers and teachers as well as a weaker tendency for the reverse, with self‐reported victims being reported by peers as aggressors. Both findings suggest that it may sometimes be difficult for peers and teachers to recognize who started the aggressive interaction. Young children may be more aware of their own experiences and less sensitive to the difficulties of others.

The findings indicate that reciprocated aggression (i.e., two children victimizing each other) was quite high, with average percentages of 15%, 20%, and 38% for self, peer, and teacher reports, respectively. In addition, a large number of children was involved in at least one victim‐aggressor relation; percentages of children that were involved varied from 68% (teacher report) to 76% (self‐reports) and 84% (peer reports).

### 4.2 Child characteristics related to informants’ reports

Victim‐aggressor relationships were different for boys and girls, and results were generally in line with previous findings in elementary schools (e.g., Veenstra et al., 2007). Self and peer reports identified boys more often as aggressors than girls, and boys harassed both boys and girls. Contrary to *H3*, girls were reported to target boys and girls to a similar extent. Teacher reports, however, showed a different pattern. Teachers reported mostly same‐sex victimization and less cross‐sex victimization, a finding also reported in a descriptive study in elementary schools (Ahn et al., 2013). Teachers may observe victimization within the context of the most common interaction patterns in kindergartens, and these are often sex‐segregated in early childhood (Cherney & London, 2006). As a consequence, victimization crossing sex‐boundaries appears not to be as salient to teachers because they may consider negative interactions between boys and girls as common behavior. However, differences were observed between teachers in the extent to which they reported cross‐sex victimization. In general, recognizing victimization is associated with teacher characteristics such as experience with and attitudes toward victimization (Oldenburg, van Duijn, et al., 2015).

Teacher reports were also differently related to parent‐reported internalizing and externalizing behavior than self and peer reports. Internalizing behavior was not found to be related to teacher reports of victimization and aggression, but with self and peer reports, children higher on internalizing behavior were less likely to be mentioned as aggressors. Contrary to our expectations (*H4b*), internalizing behavior was not found to be associated with victimization, which might be explained by low agreement between parents and teachers (and perhaps also children) in terms of internalizing symptoms, as they observe children in different contexts (Perren et al., 2006). Externalizing behavior was associated with aggression using self and peer reports, and teacher reports related externalizing behavior to both aggression and victimization (supporting *H4a*). This suggests that teachers may observe more readily victimization by children with externalizing behavior, which is likely more visible than victimization for passive (withdrawn) victims with internalizing behavior (Dawes et al., 2017). We did not find evidence for additional differences between victims and their specific aggressors regarding internalizing and externalizing behavior (contrasting *H5b*). Interpreting these findings together, aggressors were generally found to be low on internalizing behavior (e.g., being withdrawn, depressed) when identified using self and peer reports, and high on externalizing behavior (e.g., being more aggressive) when identified using self and teacher reports.

### 4.3 Limitations and strengths

Our measure of victim‐aggressor relationships did not provide information about the severity or frequency of the victimization, which might impact the opportunity to observe victimization. Second, we could not distinguish boy–girl from girl–boy victimization relations in the network models (models would not converge with a larger number of effects included). Third, the perspectives of only two informants were compared in the multivariate analyses because examination of two different networks simultaneously is currently the maximum for the available software. It would be interesting to compare the three perspectives in one network model, although this would increase the number of possible cross‐network comparisons substantively. Fourth, 40% of the parents did not respond to the questionnaire, which was solved with multiple imputations at the child level and estimating the network models five times with the different imputed datasets. Fifth, although the network approach accounted for the dependencies of victimization relations in the peer group, we did not account for other important roles in groups, such as followers, defenders, and outsiders (Belacchi & Farina, 2010; Camodeca et al., 2015; Huitsing & Monks, 2018). Future research may address also the concordance for these other roles.

Strengths of this study are the use of extensive data: 402 young children in 25 classrooms were each individually interviewed about victimization relationships (instead of only investigating individual involvement), and additionally, teacher‐ and parent‐reported data were used. The data on internalizing and externalizing behavior stemmed from different sources than data on victim‐aggressor relationships. However, we were not able to take into account that children may behave differently in different contexts. Generally, a multi‐informant approach, rather than a single‐informant approach, provides a more complete perspective on internalizing and externalizing behavior (Ladd & Kochenderfer‐Ladd, 2002; Perren et al., 2006).

### 4.4 Implications

The findings of this study may have implications for our understanding of reports on aggression in early childhood. Teachers and children report differently on victim‐aggressor relations. Compared with children's reports, teachers reported on average more girl–girl aggression and less cross‐sex aggression than children. Teachers sometimes saw the roles of victims and aggressors reversed (when compared with self‐reports), and reported more reciprocated aggression than children. Teachers also reported more victimization among children with externalizing behavior. This suggest that teachers are more likely to consider aggressive victims as victims than they are to consider passive victims (victims with mainly internalizing behavior) as being victimized. Aggressive victims may retaliate more often, which may be more visible to teachers. Because there is no consensus on an objective measure of victimization, the views of each informant should be taken seriously for signaling children at risk (Oldenburg, Barrera, et al., 2015).

In addition, practical implications follow from the study findings. First, teachers should be aware that they observe victimization differently than children; if children report victimization, teachers may not perceive their negative experiences as problematic. Second, intervention programs should pay attention to cross‐sex interactions. Interventions that focus specifically on same‐sex interactions may not be sufficient to target complete group processes. Third, parental information may be useful for the identification of at‐risk children. The current findings demonstrate that parental information on externalizing behavior was associated with children's involvement in aggression. Parents observe the behavior of their children at home and in interaction with other children outside the school context, which may be complementary to the children's behavior in the peer group. Fourth, further research may benefit from the relational perspective on victimization, because it provides more detailed information about victimization in the group context. Mutual sharing of information between parents, teachers, and children may lead to a more complete picture that contributes to signaling the development of problem behavior in the early school years.

## ETHICS STATEMENT

All procedures performed in studies involving human participants were in accordance with the ethical standards of the institutional and/or national research committee and with the 1964 Helsinki declaration and its later amendments or comparable ethical standards. Teachers were asked to participate and parents were asked permission for their children's participation. Following legal advice obtained by the Department of Education of the Canton of Bern, passive consent was obtained from parents.

## CONFLICTS OF INTEREST

The authors report no conflict of interest.
